# Energy-Efficient Resource Allocation for Near-Field MIMO Communication Networks

**DOI:** 10.3390/s25144293

**Published:** 2025-07-10

**Authors:** Tong Lin, Jianyue Zhu, Junfan Zhu, Yaqin Xie, Yao Xu, Xiao Chen

**Affiliations:** 1 School of Electronic and Information Engineering, Nanjing University of Information Science and Technology, Nanjing 210044, China; 202412490664@nuist.edu.cn (T.L.); 202283270236@nuist.edu.cn (J.Z.); xyq@nuist.edu.cn (Y.X.); yaoxu@nuist.edu.cn (Y.X.); 2 School of Artificial Intelligence, Nanjing University of Information Science and Technology, Nanjing 210044, China; x.chen@nuist.edu.cn

**Keywords:** near-field communication, massive MIMO, energy efficiency

## Abstract

With the rapid development of sixth-generation (6G) wireless networks and large-scale multiple-input multiple-output (MIMO) technology, the number of antennas deployed at base stations (BSs) has increased significantly, resulting in a high probability that users are in the near-field region. Note that it is difficult for the traditional far-field plane-wave model to meet the demand for high-precision beamforming in the near-field region. In this paper, we jointly optimize the power and the number of antennas to achieve the maximum energy efficiency for the users located in the near-field region. Particularly, this paper considers the resolution constraint in the formulated optimization problem, which is designed to guarantee that interference between users can be neglected. A low-complexity optimization algorithm is proposed to realize the joint optimization of power and antenna number. Specifically, the near-field resolution constraint is first simplified to a polynomial inequality using the Fresnel approximation. Then the fractional objective of maximizing energy efficiency is transformed into a convex optimization subproblem via the Dinkelbach algorithm, and the power allocation is solved for a fixed number of antennas. Finally, the number of antennas is integrally optimized with monotonicity analysis. The simulation results show that the proposed method can significantly improve the system energy efficiency and reduce the antenna overhead under different resolution thresholds, user angles, and distance configurations, which provides a practical reference for the design of green and low-carbon near-field communication systems.

## 1. Introduction

With the advent of sixth-generation (6G) wireless networks, massive multiple-input multiple-output (MIMO) technology has become essential for high data rate-demanding applications thanks to its high spectral efficiency and spatial resolution [1,2]. By deploying hundreds or even thousands of antennas at base stations (BSs), the Rayleigh distance can extend to several hundred meters [3,4]. Consequently, users are more likely to be in the near-field region, making traditional far-field communication models based on plane waves inapplicable [5]. Near-field communication requires a spherical-wave channel model, where channel characteristics depend on both the angle and distance of the user, providing new degrees of freedom for beamforming. Compared with traditional communication scenarios, near-field communication can fully utilize these distinctive characteristics, offering significantly more degrees of freedom and flexibility for the design of communication systems [6,7]. As wireless communication systems continue to evolve towards higher data rates and more efficient connectivity, the unique characteristics of the near-field region have emerged as a promising area for exploration.

In recent years, near-field communication has attracted significant attention from many researchers, and related research has been carried out from multiple aspects. For instance, in the area of channel modeling, works such as [2,6,8,9,10] have focused on developing accurate models for near-field channels, considering factors like spherical-wave propagation and spatial non-stationarities, which are distinct from the far-field plane-wave assumption. These models are crucial for understanding the behavior of signals in the near-field and form the basis for subsequent system design. In terms of performance analysis, some studies have evaluated the achievable data rates, energy efficiency, and reliability of near-field communication systems [5,9,11], considering both theoretical analysis and practical deployment challenges. Additionally, the integration of new technologies with near-field communication has been explored. For example, Zhang et al. investigated the combination of near-field communication with massive MIMO systems, aiming to leverage the large number of antennas in MIMO to enhance the performance of near-field communication [12,13]. Research in [14,15,16] focused on the use of intelligent reflecting surfaces (IRSs) and extremely large-scale reconfigurable intelligent surfaces (XL-RISs) in near-field scenarios to improve system performance.

However, many studies adopt single-user models for performance optimization, thereby neglecting inter-user interference. For example, in [17], authors focused on deriving lower bounds of positioning accuracy for single users or single targets without considering inter-user signal interference. Liu et al. in [2] primarily investigated near-field propagation characteristics, considering only single-user or point-to-point communication scenarios. The authors in [18] centered on the reconfigurable intelligent surface (RIS)-enhanced performance of single-user links, without addressing inter-user interference in multi-user environments. In [5], Zhang et al. derived the sum-rate expression for near-field downlink multi-user systems under the assumption of perfect focusing and no interference.

Moreover, to address inter-user interference, some authors have adopted novel multiple-access techniques to mitigate interference. For example, a flexible rate-splitting multiple-access (RSMA) framework for near-field scenarios was proposed in [19,20], where joint beam scheduling and power allocation enable the dynamic separation of private and common message components. This flexible message structure enables partial interference decoding and cancellation at the receivers. In addition, Zhang et al. [12] and Ding et al. [21] investigated the use of non-orthogonal multiple access (NOMA) in near-field or hybrid near-field and far-field environments. Through power-domain multiplexing, NOMA allows users to share spatial resources while maintaining separability through successive interference cancellation (SIC). Recent works, such as RSMA for enhanced ultra-reliable low-latency communication (URLLC) and enhanced mobile broadband (eMBB) services [22], hybrid NOMA strategies based on beam resolution [23], near- or far-field massive MIMO-NOMA connectivity optimization [24], and mmWave Near-field-NOMA beamforming [25] further advance practical interference management. These approaches can effectively manage interference when multiple users occupy overlapping spatial or angular regions.

Meanwhile, some studies have addressed inter-user interference through advanced beamforming and power control techniques. Xu et al. [26] and Yang et al. [27] highlighted the role of precoding and distributed power control in near-field and cell-free XL-MIMO systems, where beam patterns must account for spherical-wave propagation and user distance. A joint antenna selection and power allocation framework was proposed in [28], aiming to balance energy efficiency and interference suppression. These methods focus on interference-sensitive resource allocation, offering actionable optimization tools for practical applications. The use of reconfigurable antennas has emerged as a promising direction to address interference in near-field communication. Ding et al. [29] and Zhu et al. [30] proposed systems with movable antenna elements, which can dynamically adjust their spatial positions to optimize beam focus and reduce user overlap in the Fresnel region. Additionally, in [12], the authors explored dynamic metasurface antennas that adaptively manipulate the electromagnetic response to create interference-resistant near-field patterns. These techniques offer hardware-level flexibility to control interference without relying purely on signal processing.

However, the above-mentioned interference cancellation schemes impose high requirements on hardware and require high computational complexity. Therefore, in this paper, we propose an integrated optimization framework that jointly optimizes transmit power and antenna number to maximize energy efficiency by considering a resolution constraint, which ensures that interference between users can be neglected. The main contributions are summarized as follows:

- We formulate a joint optimization problem for the design of transmit power and antenna number to maximize the energy efficiency of the system, incorporating near-field beamforming resolution constraints to mitigate inter-user interference.
- Particularly, we propose a detailed analysis of the resolution constraints. Utilizing the Fresnel approximation and Taylor series expansion, a closed-form expression for the near-field resolution parameter Δ is derived to reduce the analytical complexity. By simplifying the resolution parameter, the resolution constraints in the formulated optimization problem can be effectively addressed.
- To address this optimization problem, we iteratively optimize the transmit power and antenna number and propose a two-stage alternating optimization algorithm. In the first stage, with a given number of antennas, we transform the power allocation subproblem into a convex problem via the Dinkelbach algorithm. Then, based on the optimized power allocation, we further utilize the monotonicity of the objective function to determine the optimized number of antennas in closed form.
- Finally, simulation results demonstrate the significant impact of the near-field beamforming resolution threshold on energy efficiency and the optimized number of antennas.

This paper is organized as follows. Section 2 outlines the near-field spherical-wave channel model and formulates the energy efficiency maximization problem. Section 3 analyzes the resolution of users and presents the proposed joint optimization algorithm. Section 4 evaluates performance through simulations. Section 5 concludes this paper and discusses future research directions.

Bold lowercase and uppercase letters, such as a and A, represent vectors and matrices, respectively. E[·] is the expectation of a matrix. ${\left(\cdot \right)}^{T}$,${\left(\cdot \right)}^{H}$, and ${\left(\cdot \right)}^{-1}$ denote the transpose, conjugate transpose, and inverse of a matrix, respectively. |·| indicates the absolute value.

## 2. System Model and Problem Formulation

### 2.1. System Model

**Figure 1:**
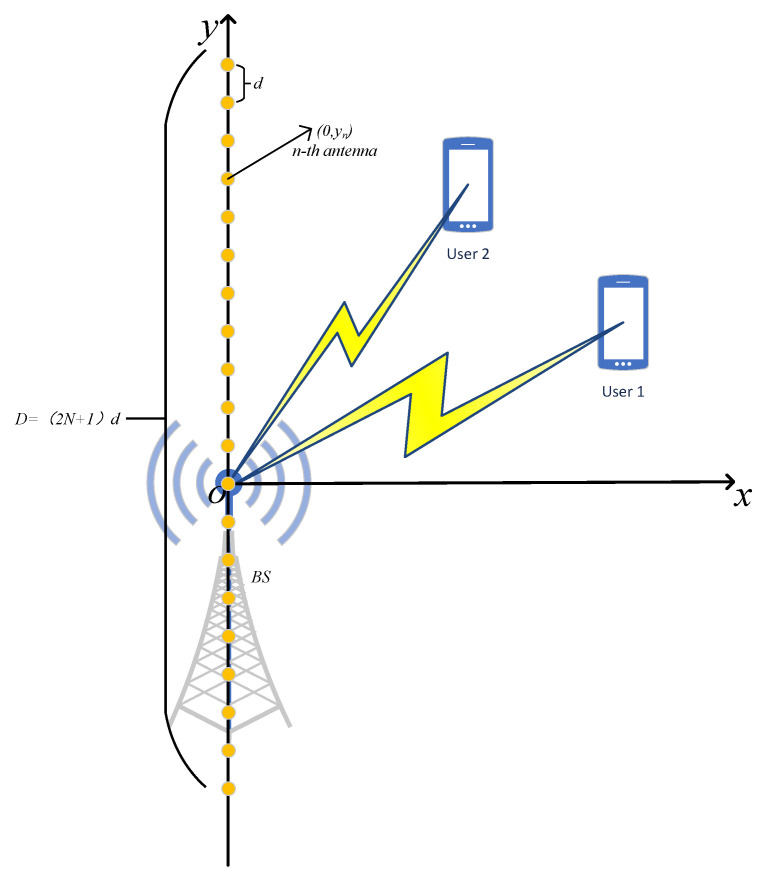
ULA-based downlink MISO communication system.

As shown in Figure 1, we consider a downlink multiple-input single-output (MISO) communication system, wherein a multi-antenna base station (BS) serves two single-antenna users. The BS is equipped with a 2N+1-antenna uniform linear array (ULA), and both the BS and the two users are assumed to be located in a two-dimensional coordinate system. Specifically, the number of antennas at the BS is assumed to be sufficiently large, such that the Rayleigh distance can reach hundreds of meters, implying that the near-field effect cannot be neglected. Therefore, this work focuses on transmission design for the near-field region. Traditionally, signal transmission has been designed for the far-field region, where the plane-wave model is employed. In contrast, for the near-field region, a more complex spherical-wave model must be applied [2]. As described in Figure 1, the Rayleigh distance is given by $d_{R}=\frac{2D^{2}}{\lambda }$, where D=(2N+1)d denotes the aperture of the ULA. Here, $d=\frac{\lambda }{2}$ denotes the antenna spacing and λ represents the carrier wavelength [3].

In this system, the transmitted signal by the BS is given by

(1) $$x=\sqrt{P_{1}}w_{1}s_{1}+\sqrt{P_{2}}w_{2}s_{2},$$

where $P_{i},i\in \left\{1,2\right\}$, denotes the transmit power allocated to the *i*-th user, and $s_{i}$ represents the normalized information symbol for the *i*-th user with $E[|s_{i}{|}^{2}]=1$. In (1), the beamforming vector is characterized by the near-field steering vector using the maximum ratio transmission (MRT) scheme, i.e., $w_{i}=b\left(r_{i}\right)$, which is given by

(2) $$\begin{array}{c} b\left(r_{i}\right)=\frac{1}{\sqrt{2N+1}}{\left[e^{-j\frac{2\pi }{\lambda }\left|r_{i}-r_{i}^{\left(-N\right)}\right|},\ldots ,e^{-j\frac{2\pi }{\lambda }\left|r_{i}-r_{i}^{\left(N\right)}\right|}\right]}^{T}, \end{array}$$

where $r_{i}=\left(r_{i},\theta_{i}\right),i\in \left\{1,2\right\}$, denotes the polar coordinates of the *i*-th user and $r_{i}^{\left(n\right)}=\sqrt{{\left(r_{i}\cos\theta_{i}\right)}^{2}+{\left(r_{i}\sin\theta_{i}-y_{n}\right)}^{2}},n\in \left[-N,N\right]$, calculates the distance between the *n*-th antenna and the *i*-th user, with $y_{n}$ denoting the coordinate of the *n*-th antenna on the *y* axis. Hence, the signal that *i*-th user receives from the BS can be given by

(3) $$\begin{array}{c} y_{i}=h_{i}^{H}x+n_{i},i\in \left\{1,2\right\}, \end{array}$$

where $n_{i}∼\mathrm{CN}\left(0,\sigma^{2}\right)$ denotes the additive Gaussian noise with power $\sigma^{2},h_{i}=\alpha_{i}b\left(r_{i}\right)$ with $\alpha_{i}=\frac{\sqrt{2N+1}\lambda }{4\pi r_{i}}$. Thus the data rate of *i*-th user to decode the signal is expressed as

(4) $$\begin{array}{c} R_{i}=\log(1+\frac{P_{i}{\left|h_{i}^{H}w_{i}\right|}^{2}}{\sum_{l\neq i}P_{l}{\left|h_{i}^{H}w_{l}\right|}^{2}+\sigma^{2}}),i,l\in \left\{1,2\right\}. \end{array}$$

Note that, different from conventional far-field communication, near-field communication is able to distinguish users through the angle domain. In order to fully utilize the characteristic of near-field transmission, we define $\Delta ≜|b{\left(r_{1}\right)}^{H}b\left(r_{2}\right){|}^{2}$ as the resolution of near-field beamforming [31]. The resolution is considered ideal when Δ≤ν, where ν is a sufficiently small threshold. Under this condition, the data rate of the *i*-th user is simplified as

(5) $$\begin{array}{c} R_{i}=\log\left(1+\frac{P_{i}{\left|h_{i}^{H}w_{i}\right|}^{2}}{\sigma^{2}}\right),i\in \left\{1,2\right\}. \end{array}$$

### 2.2. Problem Formulation

With the rapid growth of wireless services, wireless communication systems face significant challenges in achieving sustainable energy consumption. Hence, research on energy efficiency is of paramount importance for enabling green wireless communications and reducing environmental impact [32]. In this work, we focus on maximizing energy efficiency through the joint optimization of the number of antennas and power allocation. The corresponding optimization problem is formulated as follows.

(6) $$\begin{array}{cc} \max_{N\in Z^{+},P_{1},P_{2}}\; & \eta =\frac{R_{1}+R_{2}}{P_{1}+P_{2}+\left(2N+1\right)p_{f}}, \end{array}$$

(6a) $$\begin{array}{cc} s.t.\; & C1:R_{1},R_{2}\geq R_{\min}, \end{array}$$

(6b) $$\begin{array}{cc} \; & C2:P_{1}+P_{2}\leq P_{t}, \end{array}$$

(6c) $$\begin{array}{cc} \; & C3:\Delta \leq \nu . \end{array}$$

In problem (6), the energy efficiency of the system, denoted as η, is defined as the ratio of the total achievable sum data rate of all users to the overall power consumption, including both transmission power and hardware power. The parameter $p_{f}$ denotes the fixed power consumption associated with each active antenna element. Constraint C1 ensures that each user achieves a minimum data rate $R_{\min}$, thereby satisfying its individual quality-of-service (QoS) requirement. Constraint C2 limits the total transmit power to a predefined budget $P_{t}$. Constraint C3 guarantees interference-free communication between the two users by incorporating beamforming resolution limitations into the optimization process.

Problem (6) poses a challenging non-convex optimization due to its fractional objective function and the complex resolution constraint C3. First, to address this complex constraint, we perform analysis and simplification on the near-field beamforming resolution. Second, we address the energy efficiency maximization problem through iterative optimization methods.

## 3. The Joint Design of Power and Antenna Number

In this section, considering the difficulty of the formulated problem (6) partly lies in the resolution constraint, we first perform a theoretical analysis of the resolution constraint, i.e., constraint C3, and then we propose a joint alternating optimization algorithm to maximize the energy efficiency.

### 3.1. The Analysis of the Near-Field Beamforming Resolution

The resolution of near-field beamforming is given by

(7) $$\begin{array}{cc} \Delta = & |b{\left(r_{1}\right)}^{H}b\left(r_{2}\right){|}^{2} \end{array}$$

(8) $$\begin{array}{cc} = & \frac{1}{{\left(2N+1\right)}^{2}}|\sum_{n=-N}^{N}e^{-j\frac{2\pi }{\lambda }\left(|r_{1}-r_{1}^{\left(n\right)}\left|-\right|r_{2}-r_{2}^{\left(n\right)}|\right)}{|}^{2}. \end{array}$$

It can be observed that the expression in the formulation (8) is relatively complex, making it difficult to discern the relationship between resolution and other parameters, such as the number of antennas. In the following proposition, by using the Fresnel approximation and the Taylor series expansion of the cosine function, we write (8) in another form, which more easily illustrates the relationship between the near-field beamforming resolution and the number of antennas.

**Proposition** **1.** *The expression for resolution can be approximated using the following function*

(9) $$\Delta \approx 1-\frac{4}{45}\pi^{2}z^{2}N\left(4N^{3}+8N^{2}+N-3\right)-\frac{4}{3}\pi^{2}bN\left(N-1\right),$$

*where $z=\frac{\lambda }{8}\left(\frac{1-\sin^{2}\theta_{1}}{r_{1}}-\frac{1-\sin^{2}\theta_{2}}{r_{2}}\right)=\frac{\lambda }{8}\left(\frac{\cos^{2}\theta_{1}}{r_{1}}-\frac{\cos^{2}\theta_{1}}{r_{2}}\right)$ and $b=\frac{1}{2}\left(\cos\theta_{1}-\cos\theta_{2}\right)$.*

**Proof.**  Please refer to Appendix A.    □

According to Proposition 1, the expression of resolution depends only on the number of antennas and the user’s location. Moreover, for the different cases of angles of departure (AoDs), we propose the following Propositions 2 and 3 to further simplify the resolution function.

**Proposition** **2.**  *For $\theta_{1}=\theta_{2}=\theta$, the constraint C3 can be rewritten as*

(10) $$\begin{array}{c} N\left(2N+3\right)\left(2N-1\right)\left(N+1\right)\geq \frac{45(1-\nu )}{4\pi^{2}z^{2}}. \end{array}$$

*The function f(N)=N(2N+3)(2N−1)(N+1) is strictly monotonic over positive integers.*

**Proof.**  Please refer to Appendix B.    □

According to Proposition 2, when the user angles are the same, the constraint (6c) can be easily solved using numerical methods. In the next proposition, we will further describe the resolution function of different AoDs.

**Proposition** **3.** *For $\theta_{1}\neq \theta_{2}$, the constraint C3 can be rewritten as*

(11) $$\begin{array}{c} z^{2}N\left(4N^{3}+8N^{2}+N-3\right)+\frac{15}{2}\left(\cos\theta_{1}-\cos\theta_{2}\right)N\left(N-1\right)\geq \frac{45(1-\nu )}{4\pi^{2}}. \end{array}$$

*The function $g\left(N\right)=z^{2}N\left(4N^{3}+8N^{2}+N-3\right)+\frac{15}{2}\left(\cos\theta_{1}-\cos\theta_{2}\right)N\left(N-1\right)$ is strictly monotonic over positive integers.*

**Proof.**  Please refer to Appendix C.    □

By using Propositions 2 and 3, the value of *N* under constraint C3 can be obtained by numerical methods. Therefore, the simplification of the constraint C3 by Propositions 2 and 3 will facilitate a more efficient solution to the optimization problem (6).

### 3.2. The Optimization of Power to Maximize Energy Efficiency

In this subsection, we will first optimize the power with the given antenna number. The corresponding optimization problem can be given by

(12) $$\begin{array}{cc} \max_{P_{1},P_{2}}\; & \eta =\frac{R_{1}+R_{2}}{P_{1}+P_{2}+\left(2N+1\right)p_{f}}, \end{array}$$

(12a) $$\begin{array}{cc} s.t.\; & C1:R_{1},R_{2}\geq R_{\min}, \end{array}$$

(12b) $$\begin{array}{cc} \; & C2:P_{1}+P_{2}\leq P_{t}. \end{array}$$

Since problem (12) has a fractional objective, we apply the Dinkelbach transformation to convert it into an equivalent subtractive form, enabling tractable convex optimization [33,34]. As a result, we introduce the following problem

(13) $$\begin{array}{cc} \max_{P_{1},P_{2}}\; & Q_{1}\left(P_{1},P_{2},\alpha \right)≜\left(R_{1}+R_{2}\right)-\alpha [P_{1}+P_{2}+\left(2N_{0}+1\right)p_{f}, \end{array}$$

(13a) $$\begin{array}{cc} s.t.\; & C1:P_{1},P_{2}\geq \frac{16\pi^{2}r_{i}^{2}\sigma^{2}}{\left(2N+1\right))\lambda^{2}}\left(2^{R_{\min}}-1\right),i\in \left\{1,2\right\}, \end{array}$$

(13b) $$\begin{array}{cc} \; & C2:P_{1}+P_{2}\leq P_{t}. \end{array}$$

where α is the positive parameter to be updated. Lemma 1 is introduced to explain the relationship between problem (6) and problem (12).

**Lemma** **1.** Let $Q^{*}\left(\alpha \right)$ be the optimal objective value of problem (13) and $q^{*}≜\left(P_{1}^{*},P_{2}^{*}\right)$ be the optimal solution of problem (13). Then $q^{*}$ is the optimal solution to problem (12) if and only if $Q^{*}\left(\alpha \right)=0$ [33].

Obviously, problem (13) is convex and can be efficiently solved using convex optimization tools such as CVX. The optimal solution is obtained iteratively by updating the parameter α until convergence, as summarized in Algorithm 1.
**Algorithm 1** The optimization of power allocation.**Require:** Initial value $\alpha_{\mathrm{ini}}=0$, $N_{0}=10$, precision ϵ>0, constants $R_{\min}$, $P_{t}$, $p_{f}$ **Ensure:** Optimal $\alpha^{*}$ and optimal solutions $P_{1}^{*}$ and $P_{2}^{*}$
1:**Initialize:** $\alpha =\alpha_{\mathrm{ini}}$, $N=N_{0}$, $Q_{1}^{*}\left(\alpha \right)=\infty$2:**while** $|Q_{1}^{*}\left(\alpha \right)|>\epsilon$ **do**3:   Solve the optimization problem (13) with given α4:   Calculate $Q_{1}^{*}\left(\alpha \right)=\left(R_{1}^{*}+R_{2}^{*}\right)-\alpha \left[P_{1}^{*}+P_{2}^{*}+\left(2N+1\right)p_{f}\right]$5:   Update $\alpha^{*}=\eta \left(P_{1}^{*},P_{2}^{*}\right)=\frac{R_{1}^{*}+R_{2}^{*}}{P_{1}^{*}+P_{2}^{*}+\left(2N+1\right)p_{f}}$6:**end while**7:**return** $\alpha^{*}$, $P_{1}^{*}$ and $P_{2}^{*}$


### 3.3. The Optimization of Antenna Number to Maximize Energy Efficiency

In this subsection, we will further optimize the antenna number. By using the optimized power achieved by Algorithm 1, the antenna optimization problem is written as

(14) $$\begin{array}{cc} \max_{N\in Z^{+}}\; & Q_{2}\left(N,\alpha \right)≜\left(R_{1}^{*}+R_{2}^{*}\right)-\alpha \left[P_{1}^{*}+P_{2}^{*}+\left(2N+1\right)p_{f}\right], \end{array}$$

(14a) $$\begin{array}{cc} s.t.\; & C1:R_{1}^{*},R_{2}^{*}\geq R_{\min}, \end{array}$$

(14b) $$\begin{array}{cc} \; & C2:g\left(N\right)\geq \frac{45(1-\nu )}{4\pi^{2}}. \end{array}$$

where $R_{i}^{*}=\log(1+\frac{P_{i}^{*}{\left|h_{i}^{H}w_{i}\right|}^{2}}{\sigma^{2}})=\log(1+\frac{P_{i}^{*}\alpha_{i}^{2}}{\sigma^{2}}),i\in \left\{1,2\right\}$ and $g\left(N\right)=4z^{2}N^{4}+8z^{2}N^{3}+\left(z^{2}+15b\right)N^{2}-\left(3z^{2}+15b\right)N$. This problem can further be transformed into

(15) $$\begin{array}{cc} \max_{N\in Z^{+}}\; & Q_{2}\left(N,\alpha \right)=\left(R_{1}^{*}+R_{2}^{*}\right)-\alpha \left[P_{1}^{*}+P_{2}^{*}+\left(2N+1\right)p_{f}\right], \end{array}$$

(15a) $$\begin{array}{cc} s.t.\; & C1:N\geq \frac{8\pi^{2}r_{i}^{2}\sigma^{2}}{P_{i}\lambda^{2}}\left(2^{R_{\min}}-1\right)-\frac{1}{2},i\in \left\{1,2\right\}, \end{array}$$

(15b) $$\begin{array}{cc} \; & C2:N\geq t_{1}. \end{array}$$

where $t_{1}=\min\left\{N\in Z^{+}\;|\;g\left(N\right)\geq \frac{45(1-\nu )}{4\pi^{2}}\right\}$. It is evident that this is a convex problem, and the optimized solution to this problem is provided in the following Proposition 4.

**Proposition** **4.** *The close-form optimized solution to problem (* 15 *) is*

(16) $$\begin{array}{c} N=\min\left\{\left\lceil \frac{8\pi^{2}r_{i}^{2}\sigma^{2}}{P_{i}\lambda^{2}}\left(2^{R_{\min}}-1\right)-\frac{1}{2}\right\rceil ,\;t_{1}\right\}. \end{array}$$

**Proof.**  Please refer to Appendix D.    □

Proposition 4 provides a closed-form formula for determining the optimized number of antennas required to meet the near-field beamforming resolution constraint. Thus, the optimized solution $N^{*}$ of problem (15) can be obtained. Consequently, based on Lemma 1, the optimized solution $q^{*}=\left(P_{1}^{*},P_{2}^{*},N^{*}\right)$ to problems (13) and (14) is also the solution to the original energy efficiency maximization problem (6). The complete optimization process for problem (6) is presented in Algorithm 2.
**Algorithm 2** The alternating iteration optimization for energy efficiency.**Require:** Initial value $N_{0}$, iteration count *k*, precision ϵ>0 **Ensure:** Optimal solution $P_{1}^{*}$, $P_{2}^{*}$ and $N^{*}$
1:**Initialize:** $N=N_{0}$, k=1, $\eta^{0}=0$2:**while** $|\eta^{k}-\eta^{k-1}|<\epsilon$ **do**3:   Solve the optimization problem (13) with Algorithm 1 to obtain the optimal solution $P_{1}^{*}$ and $P_{2}^{*}$.4:   Solve the optimization problem (14) through numerical method to obtain the optimized solution $N^{*}$.5:   $\eta^{k}=\frac{R_{1}^{*}+R_{2}^{*}}{P_{1}^{*}+P_{2}^{*}+\left(2N^{*}+1\right)p_{f}}$6:   k=k+17:**end while**8:**return** $\eta^{k}$, $P_{1}^{*}$, $P_{2}^{*}$ and $N^{*}$


## 4. Simulation Results

In this section, we present a comprehensive numerical analysis of energy efficiency and evaluate the performance of the proposed alternating iteration joint optimization algorithm via simulations. In the simulations, the BS equipped with a ULA of 2N+1 antennas is placed at the cell center, while the two users are located at distances $r_{1}$ and $r_{2}$ with AoD coordinates $\theta_{1}$ and $\theta_{2}$, respectively. The total BS transmission power is set to $P_{t}$, and the fixed power consumption of $p_{f}=0.1$ W per antenna is selected based on the typical power consumption reported in [32] for analog radio frequency chains. The noise power is calculated based on the thermal noise formula, i.e., $\sigma^{2}=BN_{0}$, where B=1000MHz is the bandwidth corresponding to the expected allocation for high-throughput 6G systems [1,4], and $N_{0}=-174\;\mathrm{dBm}/\mathrm{Hz}$ is the noise power spectral density. Next, we will conduct simulations on the impacts of resolution, total power, and QoS constraints on energy efficiency, as well as the effect of resolution on the optimized number of antennas. All simulations are conducted in MATLAB R2022a using the CVX toolbox for convex optimization.

**Figure 2:**
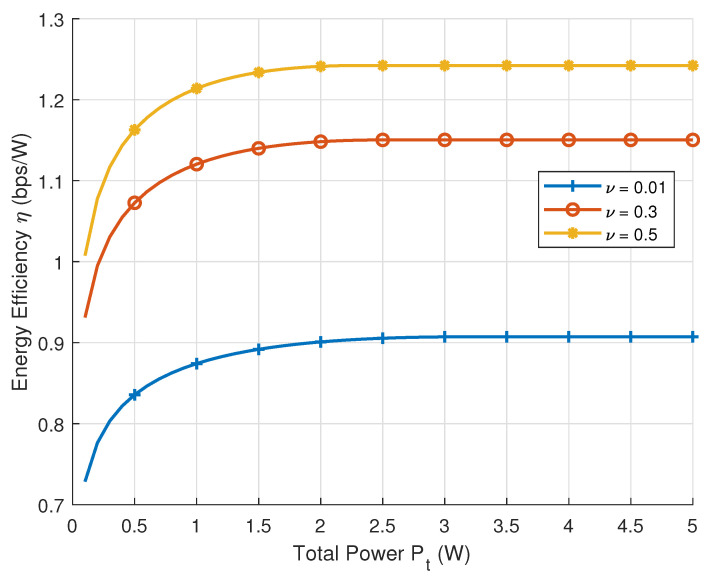
Energy efficiency versus transmission power with different resolution thresholds.

In Figure 2, we focus on the variation in energy efficiency with respect to the total transmit power, presenting a comparative analysis of energy efficiency curves under different resolution thresholds ν while keeping fixed parameters $r_{1}$, $r_{2}$, $\theta_{1}$, and $\theta_{2}$. The figure demonstrates that at low transmit power levels, the limited power resources result in the energy efficiency not reaching the optimal value, while with the increase in the transmit power, the energy efficiency gradually increases until it reaches the maximum value. Meanwhile, as the resolution threshold increases, the energy efficiency also improves due to the reduced requirement of spatial resolution, which allows fewer antennas to be deployed.

**Figure 3:**
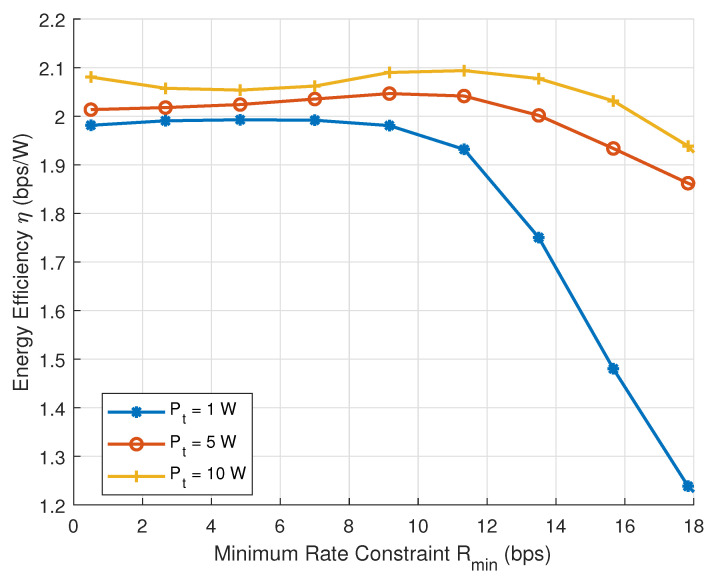
Energy efficiency versus the minimum rate thresholds with different transmission power.

Figure 3 shows the energy efficiency performance versus the minimum rate constraint $R_{\min}$ under different transmit powers. It is observed that energy efficiency remains stable at low $R_{\min}$ and drops rapidly beyond a certain threshold, particularly for low-power settings. This trend occurs because satisfying stringent QoS constraints requires increased transmission power and potentially more antennas, leading to a sharp rise in total power consumption. Furthermore, higher transmit power leads to higher user rates, reducing the number of antennas required to meet the same QoS threshold, thereby enhancing system energy efficiency.

**Figure 4:**
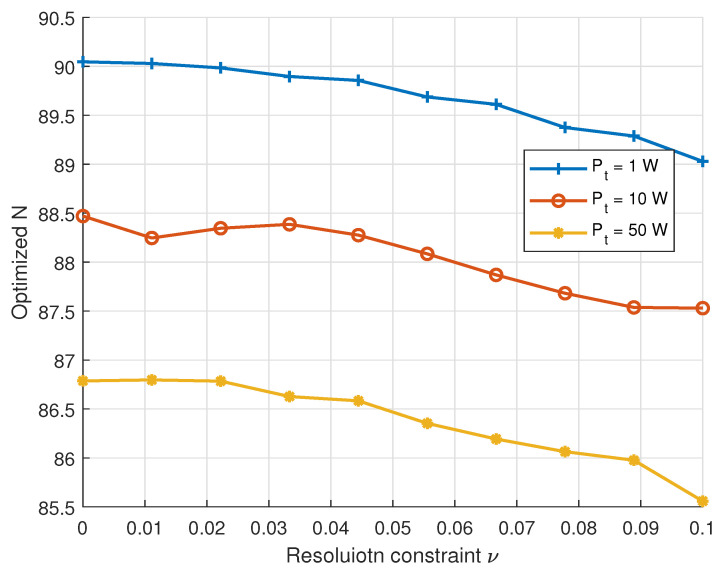
Optimized number of antennas versus resolution thresholds with different transmission power.

Figure 4 illustrates the variation in the average optimized antenna number *N* with respect to the resolution threshold ν under different total transmit powers. It is observed that *N* decreases monotonically with increasing ν, as a relaxed resolution constraint permits fewer antennas to achieve the required spatial discrimination. Moreover, higher transmit power results in lower antenna requirements, as the system can meet both rate and resolution constraints using less spatial diversity and more power. These trends are consistent with the theoretical analysis in (10) and (11), confirming the joint impact of spatial resolution and power availability on antenna configuration in near-field communication.

**Figure 5:**
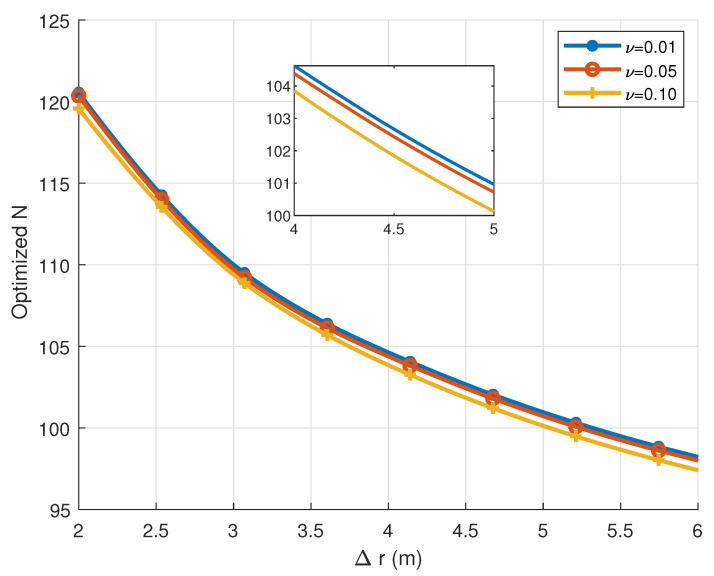
Optimized number of antennas versus the users’ distance with different resolution thresholds.

Figure 5 shows the variation in the optimized antenna number with respect to the distance difference $\Delta r=r_{2}-r_{1}$ between two users for various resolution thresholds ν. The results indicate that increasing Δr generally requires fewer antennas to effectively distinguish users. As users move farther from the BS, the channel increasingly approximates the far-field plane-wave model, enhancing user separability.

**Figure 6:**
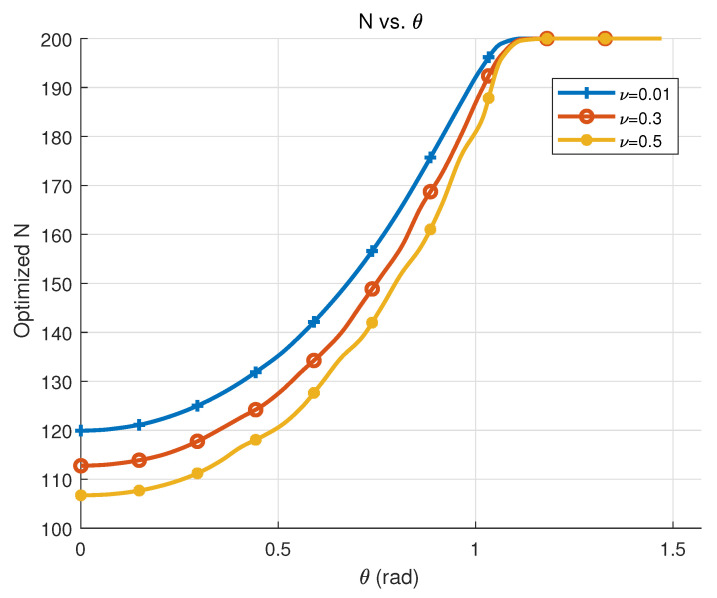
Optimized number of antennas *N* versus the same user angle $\theta =\theta_{1}=\theta_{2}$ under resolution thresholds ν.

Figure 6 displays the relationship between the optimized number of antennas *N* and the user angle under different resolution thresholds ν in the special case with fixed $r_{1}$ and $r_{2}$ and the same AoDs of two users. It can be clearly seen that the smaller θ is, the smaller the number of antennas required after optimization, and the variation in N is non-linear as θ increases. This is because the smaller theta is, the larger $z=\frac{\lambda \cos^{2}\theta }{8}\left(\frac{1}{r_{1}}-\frac{1}{r_{2}}\right)$ is, at which point the resolution constraint $\Delta \approx 1-\frac{4}{45}\pi^{2}z^{2}N\left(4N^{3}+8N^{2}+N-3\right)\leq \nu$ is satisfied by a smaller *N*.

While the optimized number of antennas in our simulations reaches approximately 100, this is considered reasonable in near-field communications. Due to the limited spatial separation and high resolution requirements, large-scale antenna arrays are necessary. Moreover, with the advancement of metasurface and dynamic array technology [12,30], such configurations are feasible and practical for future 6G deployments.

## 5. Conclusions

This paper proposed a novel energy-efficient framework for near-field communication systems by jointly optimizing antenna number and power allocation. Through the application of the Fresnel approximation and Taylor series expansion, a closed-form spatial resolution constraint was derived, enabling the reformulation of a non-convex beamforming problem into a tractable polynomial inequality. To address the formulated optimization problem, an efficient alternating algorithm was developed. Simulation results validate the effectiveness of the proposed method and reveal the impact of resolution constraints on the number of antennas and energy efficiency. These findings not only provide empirical support for the theoretical analysis but also offer significant guidance for the practical deployment of near-field massive MIMO in future 6G systems. Nevertheless, this study is constrained to a simplified two-user scenario with idealized assumptions regarding channel conditions and ideal antenna elements. Future work will extend this framework to multi-user settings and explore the integration of intelligent reconfigurable surfaces to further enhance system performance.

## Figures and Tables

**Figure A1:**
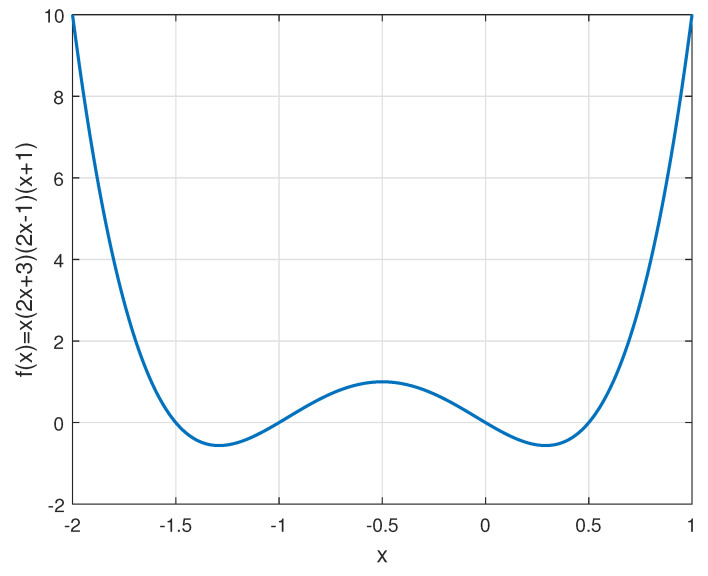
The plot of f(x)=x(2x+3)(2x−1)(x+1).

## Data Availability

The original contributions presented in this study are included in the article. Further inquiries can be directed to the corresponding author.

## Appendix A. Proof of Proposition 1

The distance between the *n*-th antenna and the *i*-th user is

(A1) $$\begin{array}{cc} r_{i}^{\left(n\right)} & =\sqrt{{\left(r_{i}\cos\theta_{i}\right)}^{2}+{\left(r_{i}\sin\theta_{i}-y_{n}\right)}^{2}} \end{array}$$

(A2) $$\begin{array}{cc} & =r_{i}\sqrt{1+\frac{y_{n}^{2}-2r_{i}y_{n}\sin\theta_{i}}{r_{i}^{2}}}, \end{array}$$

where $y_{n}=nd=\frac{\lambda }{2}n$, n∈[−N,N] and i∈{1,2}. It is assumed that $r_{i}\gg y_{n}$, so $\frac{y_{n}^{2}-2r_{i}y_{n}\sin\theta_{i}}{r_{i}^{2}}\to 0$. Therefore, we can use the approximation $\sqrt{1+x}\approx 1+\frac{1}{2}x-\frac{1}{8}x^{2}\;\mathrm{for}\;x\to 0$. Hence the distance can be expressed as

(A3) $$\begin{array}{c} r_{i}^{\left(n\right)}\approx r_{i}-y_{n}\sin\theta_{i}+\frac{y_{n}^{2}\cos^{2}\theta_{i}}{2r_{i}}. \end{array}$$

Applying (A3) and incorporating trigonometric relationships, the resolution Δ can be expressed as follows:

(A4) $$\begin{array}{cc} \Delta & \approx \frac{1}{{\left(2N+1\right)}^{2}}|\sum_{n=-N}^{N}e^{-j\frac{2\pi }{\lambda }\left(\frac{y_{n}^{2}\cos^{2}\theta_{1}}{2r_{1}}-\frac{y_{n}^{2}\cos^{2}\theta_{2}}{2r_{2}}\right)}{|}^{2} \end{array}$$

(A5) $$\begin{array}{cc} \; & =\frac{1}{{\left(2N+1\right)}^{2}}|\sum_{n=-N}^{N}e^{-j2\pi n^{2}\frac{\lambda }{8}\left(\frac{\cos^{2}\theta_{1}}{r_{1}}-\frac{\cos^{2}\theta_{2}}{r_{2}}\right)}{|}^{2}. \end{array}$$

Letting $z=\frac{\lambda }{8}\left(\frac{\cos^{2}\theta_{1}}{r_{1}}-\frac{\cos^{2}\theta_{2}}{r_{2}}\right)$ and expanding the square of complex modulus, we can obtain the following

(A6) $$\begin{array}{cc} \Delta & =\frac{1}{{\left(2N+1\right)}^{2}}\sum_{n=-N}^{N}\sum_{k=-N}^{N}e^{j2\pi z(k^{2}-n^{2})} \end{array}$$

(A7) $$\begin{array}{cc} \; & =\frac{1}{{\left(2N+1\right)}^{2}}\sum_{n=-N}^{N}\sum_{s=-N-n}^{N-n}e^{j2\pi z({\left(n+s\right)}^{2}-n^{2})} \end{array}$$

(A8) $$\begin{array}{cc} \; & =\frac{1}{{\left(2N+1\right)}^{2}}\sum_{s=-2N}^{2N}\sum_{n=\max(-N,-N-s)}^{\min(N,N-s)}e^{j2\pi s(zs+2zn-b)}. \end{array}$$

Since $e^{j2\pi x}+e^{-j2\pi x}=2\cos\left(2\pi x\right)$, Δ can be written as follows [3]:

(A9) $$\begin{array}{cc} \Delta & =\frac{2}{{\left(2N+1\right)}^{2}}\sum_{s=0}^{2N}\sum_{n=-N}^{N-s}\cos\left[2\pi s\left(zs+2zn-b\right)\right] \end{array}$$

(A10) $$\begin{array}{cc} \; & =\frac{1}{2N+1}+\frac{2}{{\left(2N+1\right)}^{2}}\sum_{s=1}^{2N}\sum_{n=-N}^{N-s}\cos\left[2\pi s\left(zs+2zn-b\right)\right]. \end{array}$$

Now, the expression for Δ remains complex, making it challenging to derive the constraint C3 directly from its current form. To proceed with further simplification, the summation term $\sum_{n=-N}^{N-s}\cos\left[2\pi s\left(zs+2zn-b\right)\right]$ must first be addressed. If we assume *n* is a sufficiently small constant, we can approximate cos[2πs(zs+2zn−b)] using the Taylor series expansion of the cosine function. The Taylor series expansion of cosine function is as follows: $\cos\left(x\right)=1-\frac{x^{2}}{2!}+O\left(x^{3}\right)$. Substituting x=2πs(zs+2zn−b) into the series, we obtain the following $\cos\left[2\pi s\left(zs+2zn-b\right)\right]\approx 1-\frac{1}{2}{\left[2\pi s\left(zs+2zn-b\right)\right]}^{2}$. The term $O({\left[2\pi s\left(zs+2zn-b\right)\right]}^{3})$ represents a higher-order infinitesimal relative to ${\left[2\pi s\left(zs+2zn-b\right)\right]}^{2}$, and its value can be neglected when s approaches 0. After simplifying, the formula of Δ is shown in Proposition 1.

## Appendix B. Proof of Proposition 2

For $\theta_{1}=\theta_{2}$, $b=\frac{1}{2}\left(\cos\theta_{1}-\cos\theta_{2}\right)=0$. Hence the inequality C3 can be equivalent to Equation (10) Based on the continuous function

(A11) $$\begin{array}{c} f(x)=x(2x+3)(2x-1)(x+1), \end{array}$$

which has real zeros at x=−1.5,−1,0 and 0.5, we verify its monotonicity by analyzing its first derivative. The first derivative

(A12) $$\begin{array}{c} f^{\prime }\left(x\right)=16x^{3}+24x^{2}+2x-3 \end{array}$$

has three real roots x≈−1.2906,−0.5000,+0.2906, which partition the real line into alternating intervals of monotonic decrease and increase and thus yield a local minimum at x≈−1.2906, a local maximum at x=−0.5, and a second local minimum at x≈0.2906; further, the second derivative

(A13) $$\begin{array}{c} f^{\prime \prime }\left(x\right)=48x^{2}+48x+2 \end{array}$$

vanishes at x≈−0.9564 and −0.0436, marking the two inflection points where the concavity changes sign, and finally, examining the forward difference for positive integers

(A14) $$\begin{array}{c} \Delta f\left(n\right)=f\left(n+1\right)-f\left(n\right)=4\left(4n^{3}+14n^{2}+13n+3\right)>0,\;n\in Z^{+} \end{array}$$

establishes that f(n+1)>f(n) for all n≥1; hence f(x) is strictly increasing on the positive integers. These analytical results can be corroborated by plotting in MATLAB R2022a shown as Figure A1.

Since the leading term is $4x^{4}$ with a positive coefficient,

(A15) $$\begin{array}{c} \lim_{x\to \pm \infty }f\left(x\right)=+\infty . \end{array}$$

Therefore, when *N* is a positive integer, f(N) is monotonically increasing.

## Appendix C. Proof of Proposition 3

To make the inequality easier to analyze, we define $g_{1}\left(N\right)≜z^{2}f\left(N\right)=z^{2}N\left(4N^{3}+8N^{2}+N-3\right)$ and $g_{2}\left(N\right)≜15bN\left(N-1\right)$, so the inequality (11) can be expressed as

(A16) $$\begin{array}{c} g\left(N\right)\geq \frac{45(1-\nu )}{4\pi^{2}}, \end{array}$$

where $g\left(N\right)≜g_{1}\left(N\right)+g_{2}\left(N\right)$. When $\theta_{1}<\theta_{2}$, i.e., b>0, it is evident that $g_{2}\left(N\right)$ is monotonically increasing for $N\in Z^{+}$. Since $z^{2}$ is positive and does not change monotonicity, the monotonicity of $g_{1}\left(N\right)$ is the same as f(N). According to Proposition 2, $g_{1}\left(N\right)$ increases monotonically when *N* is a positive integer. Since both $g_{1}\left(N\right)$ and $g_{2}\left(N\right)$ are strictly increasing over the positive integers, their sum $g_{1}\left(N\right)+g_{2}\left(N\right)$ also increases strictly.

When $\theta_{1}>\theta_{2}$, i.e., b<0, the complete expansion of g(N) yields the following expression

(A17) $$g\left(N\right)=4z^{2}N^{4}+8z^{2}N^{3}+\left(z^{2}+15b\right)N^{2}-\left(3z^{2}+15b\right)N.$$

In order to investigate the monotonic behavior of g(N) over the set of positive integers, we analyze the function by deriving its first-order derivative. The derivative of g(N) is given by the following

(A18) $$\begin{array}{cc} g^{\prime }\left(N\right) & =16z^{2}N^{3}+24z^{2}N^{2}+\left(2z^{2}+30b\right)N-\left(3z^{2}+15b\right)\\ \; & =z^{2}\left(16N^{3}+24N^{2}+2N\right)+30bN-\left(3z^{2}+15b\right). \end{array}$$

Among the first three terms, the cubic term dominates and is positive. The term −30bN is a negative linear term. However, its growth rate is slower than that of the cubic term $N^{3}$. The constant term does not affect the overall trend. Therefore, when *N* is sufficiently large, e.g., N≥2, the derivative is guaranteed to be positive. Hence, the function g(N) exhibits monotonic growth when *N* is a positive integer.

## Appendix D. Proof of Proposition 4

Since $t_{1}$ is derived from the equivalence,

(A19) $$\begin{array}{cc} t_{1} & =\min\{N|g\left(N\right)\geq \frac{45(1-\nu )}{4\pi^{2}}\}. \end{array}$$

The increased monotonicity of *g* implies that $\left\{N|g\left(N\right)\geq \frac{45(1-\nu )}{4\pi^{2}}\right\}$ has a well-defined minimum $t_{1}$. For $N\geq t_{1}$, by definition of minimum and monotonicity, $g\left(N\right)\geq g\left(t_{1}\right)\geq \frac{45(1-\nu )}{4\pi^{2}}$. Given that the objective function is monotonically decreasing, the optimized solution *N* to problem (15) is the smallest integer value that satisfies the constraints, i.e., $\min\{\frac{8\pi^{2}r_{i}^{2}\sigma^{2}}{P_{i}\lambda^{2}}\left(2^{R_{\min}}-1\right)-\frac{1}{2},t_{1}\}$.
